# Magnetotelluric data analysis using 2D inversion: A case study from Al-Mubazzarah Geothermal Area (AMGA), Al-Ain, United Arab Emirates

**DOI:** 10.1016/j.heliyon.2021.e07440

**Published:** 2021-06-29

**Authors:** Hakim Saibi, Sadieh Khosravi, Biruk Abera Cherkose, Maxim Smirnov, Yosef Kebede, Abdel-Rahman Fowler

**Affiliations:** a Geology Department, College of Science, UAEU, Al-Ain, United Arab Emirates; b Luleå University of Technology, Sweden; c Geological Survey of Ethiopia, Ethiopia

**Keywords:** Magnetotelluric, Potential field, 2D inversion, Geothermal, AMGA, Low-enthalpy, Al-Ain, United Arab Emirates

## Abstract

Geothermal manifestations (hot springs) emerge in the Al-Mubazzarah Geothermal Area (AMGA), Al-Ain city, Abu Dhabi Emirate, United Arab Emirates. This paper presents the application and results of a Magnetotelluric (MT) survey, which was carried out in 2017 at the AMGA geothermal field. The MT method was used to investigate the variations in the electrical conductivity beneath the AMGA. This study focuses on characterizing the patterns of subsurface electrical conductivity of the AMGA geothermal reservoir. Dimensionality analysis of the measured MT data indicate that 2D inversion is appropriate for the subsurface resistivity interpretation. The inversion results support a model consisting of three resistivity-defined layers; from top to bottom they are: (1) a shallow layer with resistivity ranging from 10 to 20 Ωm, representing recent alluvial and windblown deposits, (2) a second conductive layer with resistivities less than 10 Ωm, beneath the first layer. This layer is recognized as the Tertiary carbonate sequence in the region, (3) a deep, moderate to relatively high resistive zone, 10–30 Ωm beginning at 800 m depth and reaching 4 km depth in the northern part of the profile, representing Mesozoic basement rocks. The observed moderate to high resistivity zone (10–30 Ωm) in the 2D model may represent a region where the hot groundwaters originated (geothermal reservoir), with the hottest geothermal located at a depth greater than 4 km. The geothermal reservoir zone is also represented by a low to high density contrast and a low to moderate magnetic susceptibility, as indicated in the inverted potential field data models, and confirmed the existence of a north dipping major fault.

## 1 Introduction

Al-Mubazzarah Geothermal Area (AMGA) is located in the foothill of the Jabal Hafit Mountain, around 20 km south of Al-Ain city in the United Arab Emirates. The Al-Mubazzarah area has been irrigated to develop a green area in the middle of the desert as a touristic attraction. Al-Mubazzarah is also known for its hot springs, which provide therapeutic thermal waters for baths throughout the year. The temperature of the hot spring water averages about 40 °C (Saibi et al., 2020). The sources of the hot water and the size of the hydrothermal reservoir are still unknown. This geothermal region is considered as a low enthalpy geothermal zone. Low-enthalpy geothermal systems in non-volcanic areas are usually related with hot fluids in sedimentary or crystalline reservoirs, either with natural permeability or enhanced fluid pathways (Muñoz, 2014). Low-enthalpy reservoirs have low reservoir temperature (<150 °C) compared to the high enthalpy geothermal systems, where temperatures exceed 150 °C.

Geophysical techniques, in particular the Magnetotelluric (MT) method, have been employed in investigations of subsurface geological features at geothermal fields. MT is a geophysical method that uses the Earth's naturally occurring electromagnetic fields to study the electrical properties of the subsurface. It is particularly useful in exploring for deep targets due to its deep penetration, which is not achievable with other electromagnetic methods. The results of resistivity distributions can be used to map the lithology, contacts/faults, locations of geothermal reservoirs, and groundwater aquifers. For low-enthalpy geothermal systems, the MT method has been applied in many countries and a good summary of MT applications in low-enthalpy areas can be found in a review paper by Muñoz (2014). MT has been applied in a range of areas and geological settings. A few examples of applications of the MT method in low enthalpy systems include: locating possible geothermal source zone in the Chabsar hot spring, India (Mohan et al., 2017); the study of the geothermal zone in Hamam Faraun, Egypt, in order to identify the hot spring sources (Abdel Zaher et al., 2012); and exploration of shallow geothermal fluid reservoirs for the Fang geothermal system, Thailand (Amatyakul et al., 2016). MT has been used to clarify the geological structures associated with the Gediz Graben geothermal area in Turkey, using 3-D inversion to produce a conceptual model for the resistive basement rock structure (Erdoğan and Candansayar, 2017). Other MT study areas include Gross Schönebeck in Germany (Muñoz et al., 2010) and at the Pohang Low-Enthalpy geothermal area in Korea (Uchida et al., 2005).

In general, a geothermal system consists of a source of heat, a permeable reservoir that permits water to flow freely through pore space or fractures, and an impermeable trap (cap rock) that prevents dispersion of hot water to the surface and keeps it under pressure. A number of factors affect the resistivity of the rocks including temperature, porosity, alteration, and salinity of fluid in the pore space (Cherkose and Mizunaga, 2018). However, the main factor affecting the resistivity of rocks in geothermal systems is the presence of hydrothermal alteration and its relationship to temperature (Hersir and Árnason, 2009). The MT method may be used to construct the resistivity structure of a geothermal system and also yields information about the temperature and other attributes of interest in the geothermal zones (Mohan et al., 2019). The temperature distribution causes hydrothermal rock alterations that are assisted by convection of hydrothermal waters, commonly leading to the formation of clay minerals. Differences in electrical conductivities of these alteration minerals is a significant factor in geothermal heat source prospecting (Chiragwile, 2009).

This study is the first to apply the MT method to the Al-Mubazzarah area, in the city of Al Ain in the United Arab Emirates (UAE). The main objectives of this study are to map the resistivity distribution beneath the study area, locate the geothermal reservoir, and delineate subsurface geological structures (faults) that may control the migration of geothermal fluids in this area.

## 2 Geological and geothermal settings

**Figure 1:**
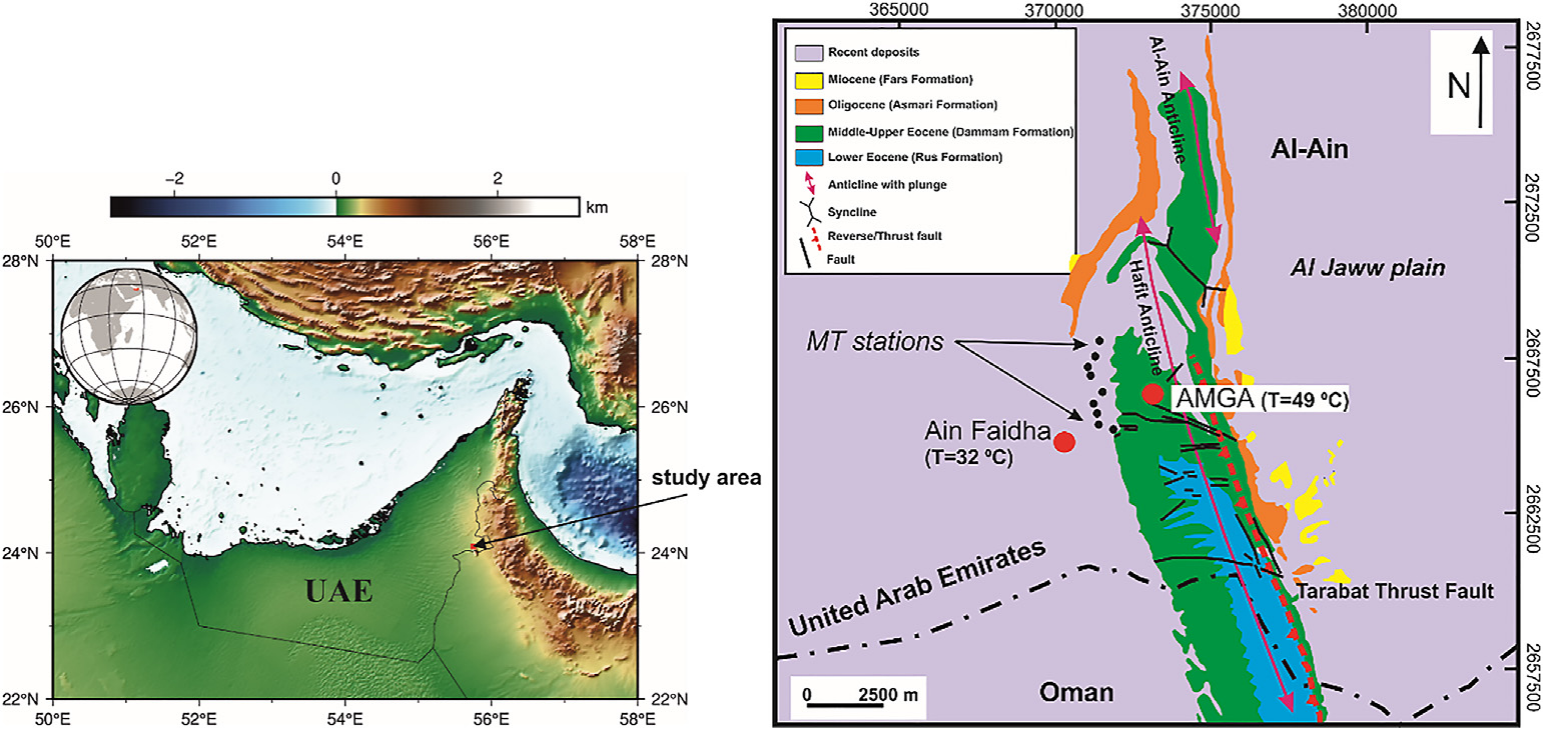
Location of the study area (Left). Geological map of the study area (Right) with the locations of AMGA and Ain Faidha hot spring. The E-W to WNW-ESE trending fractures are vertical tension fissures, partly reused as strike-slip faults. Also with approximately E-W trends are normal faults with steep N and S dips. NNW-striking faults are thrusts. NE-trending faults are strike-slip (modified from Ali et al., 2009).

Near the end of the Cretaceous, the ophiolitic rocks that now fringe the southeastern margin of the Arabian platform were transported from the ocean realm to their present position by obduction of a slice of oceanic crust and upper mantle, referred to as the Semail Ophiolite. In the Late Eocene-Miocene, the collision of the Arabian Plate with the Eurasian Plate resulted in a second compressional event that produced large-scale folding and the reactivation of deep-seated faults in the frontal fold and thrust belt and adjacent foreland basin (Boote et al., 1990; Dunne et al., 1990; Searle et al., 1990). In addition, a mainly carbonate cover sequence unconformably above the Semail Ophiolite was uplifted. One of the remarkable structures produced during this event is the Hafit Anticline lying in the area between the Northern and Central Oman Mountains, and just south of the modern city of Al-Ain (Glennie, 1974). The Jabal Hafit fold is a doubly plunging asymmetric anticline that trends NNW-SSE. This mountain is 28 km long and 4 km wide and is a prominent structure in the frontal northern Oman Mountains (Figure 1).

Jabal Hafit Mountain represents the hinge zone of the eroded Hafit Anticline. The mountain is the UAE's second highest peak, with the height of approximately 1240 m. Hafit Mountain is separated from the main northern Oman Mountain chain by the Al Jaww plain. The Tertiary units deformed by the Hafit Anticline include four defined formations, from oldest to youngest: the Rus Formation, Dammam Formation, Asmari Formation and Lower Fars Formation.

The Lower Eocene Rus Formation is exposed in the core of the anticline and contains a sequence of thin-bedded nodular cherty limestones with massive fossiliferous limestone intercalations. The Rus Formation is overlain by the Middle-Late Eocene Dammam Formation comprising a sequence of thick hard Nummulitic limestones and marls followed by thinly bedded calcarenite grainstones. Above the Dammam is the Asmari Formation of Oligocene age represented by Nummulitic limestone with coralgal bioherms and marl interbeds. Finally, the Lower Fars Formation is a Miocene unit dominated by gypsiferous sediments deposited in a lagoonal setting. The Lower Fars is unconformably covered by alluvial fan conglomerates and carbonate-cemented sands of the Barzaman Formation (Pliocene - ?Recent), that has abundant gravels of Semail-derived peridotite. The Barzaman, in turn, is covered by more recent alluvium and wind blown sands deposited since at least Pleistocene times.

All areas included the bedrock fracture systems studied by Zaineldeen and Fowler (2014) showed E-W tension fractures, which have later been used as normal faults and oblique strike-slip faults, as the dominant bedrock discontinuities (Figure 1). Styles et al. (2006) also detected many large faults with approximately E-W strikes (Figure 1). Ain Faidha hot springs area lies westward along the trend from one of these major E-W fractures that is known to supply thermal waters in the Al-Mubazzarah area. Although there is much surface cover obscuring the trace of this fracture system, there is a good possibility that it may extend into the AMGA area.

**Figure 2:**
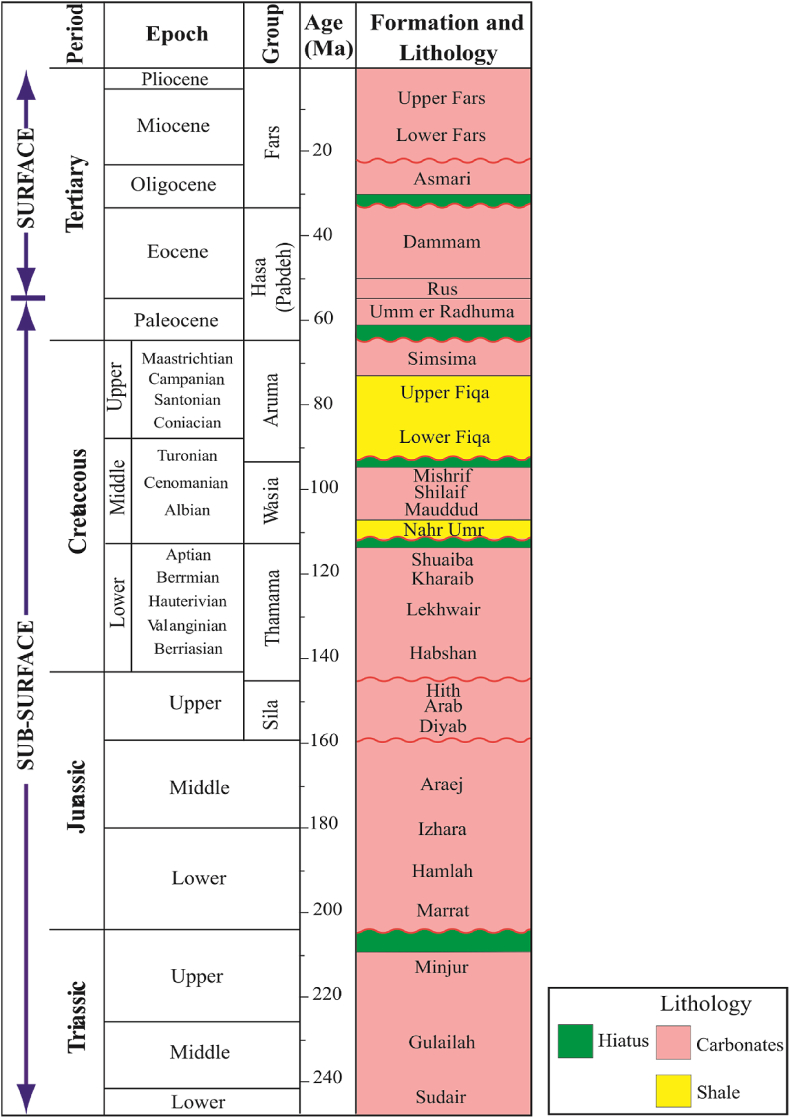
Stratigraphic log of Jabal Hafit (modified from Ali at al., 2009).

Ali et al. (2009) conducted a seismic survey across Jabal Hafit and onto the Al Jaww plain. In their interpretation of seismic profiles across the Hafit Mountain they identified the subsurface rock units as Lower Fars overlying the Tertiary units (Oligocene Asmari down to Early Eocene Rus Formations). Beneath these units, there were units not exposed anywhere in the Al Ain area that included: Late Cretaceous foreland basin sediments (Fiqa Formation) interrupted by a thrust sequence of Mesozoic deep marine Hawasina Group sediments. These were unconformably lying upon a Mesozoic carbonate shelf sequence (Figure 2).

The beds on the western limb of the Hafit anticline dip about 30° to the West, while the eastern limb shows dips varying between 70°E and 90°. The Hafit Anticline has a ~2 km wide flat crestal area (Ali et al., 2009). A westward dipping reverse fault, the Tarabat thrust fault, cuts the eastern limb of the Hafit anticline (Noweir, 2000).

The main geothermal manifestations in the study area are hot springs emerging through fault and fracture zones in the study area. Hot waters from the springs were sampled and analysed (chemistry, pH, temperature) to help determine the origin of the fluids. The discharge surface temperatures of hot waters range from 32 °C to 49 °C. The thermal waters are mainly Na-Cl water type (Saibi et al., 2021).

## 3 The magnetotelluric method

The Magnetotelluric (MT) method is a geophysical technique used to determine how the property of electrical conductivity varies in the subsurface, based on the response to natural transient electric and magnetic fields at the surface (Simpson and Bahr, 2005). MT is a passive geophysical technique that utilizes naturally occurring geomagnetic variations as the power source. In MT survey, two components of the electric field (*Ex* and *Ey*) and three components of the magnetic field (*Hx*, *Hy* and *Hz*) are measured. These natural electromagnetic fields are generated in the Earth's atmosphere mainly by lightning, and by interactions between the solar wind and the Earth's ionosphere. The MT data frequency ranges from 0.0001 Hz to 1000 Hz, which leads to the wide range of depths of penetration that can be achieved using MT methods (Simpson and Bahr, 2005). Apparent resistivity of the subsurface is determined from the relationship between the measured electric and magnetic fields as follow,

(1) $$\rho_{a}=\frac{1}{\mu_{0}\omega }{\left|z\left(\omega \right)\right|}^{2},$$

where $\;\rho_{a}$ is the apparent resistivity in Ω-m, $\mu_{0}$ is the magnetic permeability of free space (4π×10^−7^ H/m), ω is the angular frequency. And **Z** is a frequency dependent complex impedance tensor, that contains information about the subsurface conductivity structure, and is related to the measured electric (*Ex* and *Ey*) and magnetic components (*Hx* and *Hy*) in orthogonal directions, via the formula below:

(2) $$\left(\begin{array}{l} E_{x}\\ E_{y} \end{array}\right)=\left(\begin{array}{ll} Z_{xx}\; & Z_{xy}\\ Z_{yx}\; & Z_{yy} \end{array}\right)\left(\begin{array}{l} H_{x}\\ H_{y} \end{array}\right),$$

Where *Zxx*, *Zxy*, *Zyx* and *Zyy* are components of the impedance tensor (**Z**).

The frequency (inverse of period) of the electromagnetic signals used in MT, along with the electrical resistivity of the subsurface, determines how deeply the signal propagates in the subsurface.

### 3.1 MT survey at AMGA

**Figure 3:**
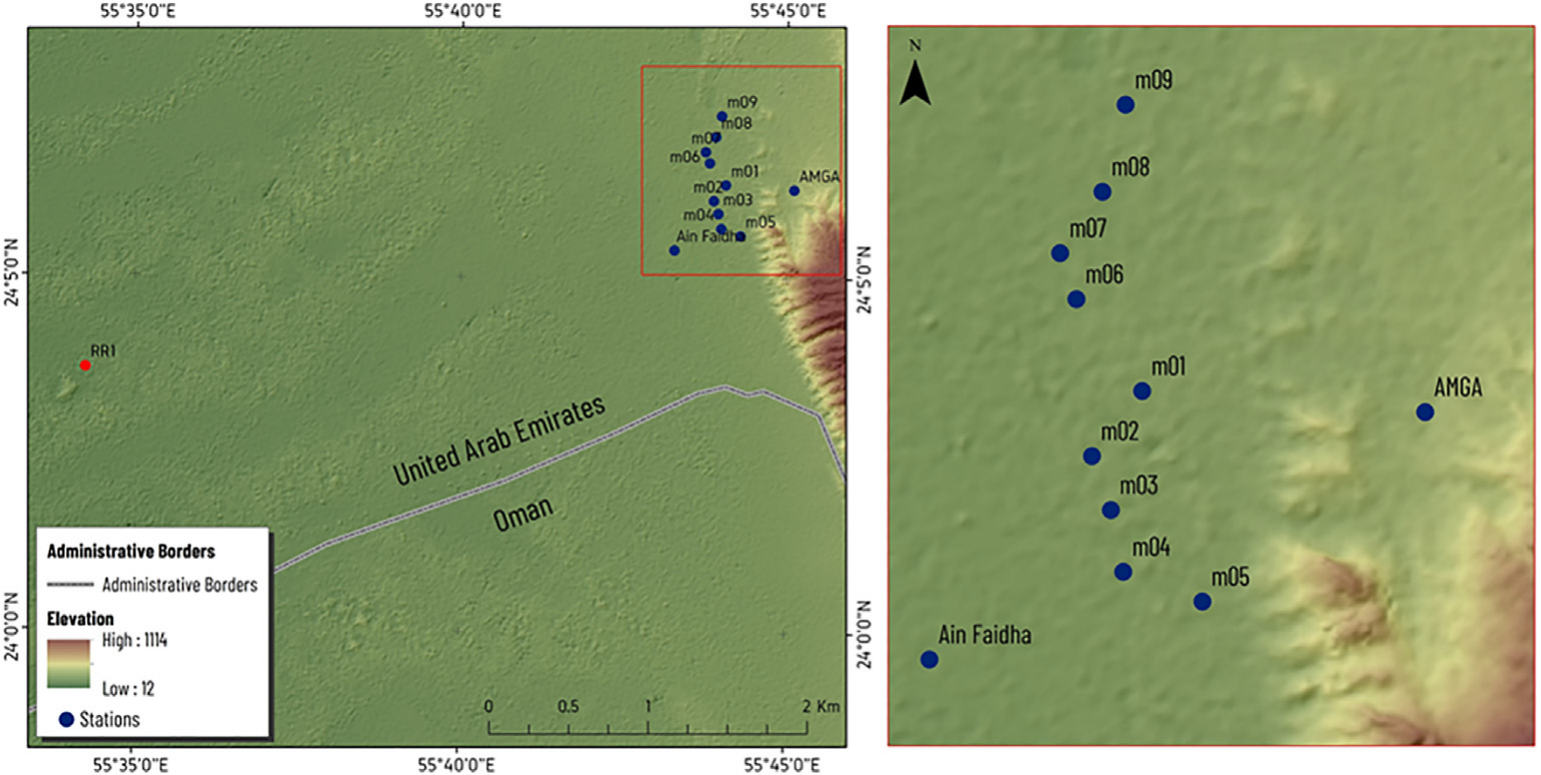
Location Map of Al-Mubazzarah Geothermal Area (AMGA) showing MT Station points (m01-m09) and RR1 is the reference station.

**Figure 4:**
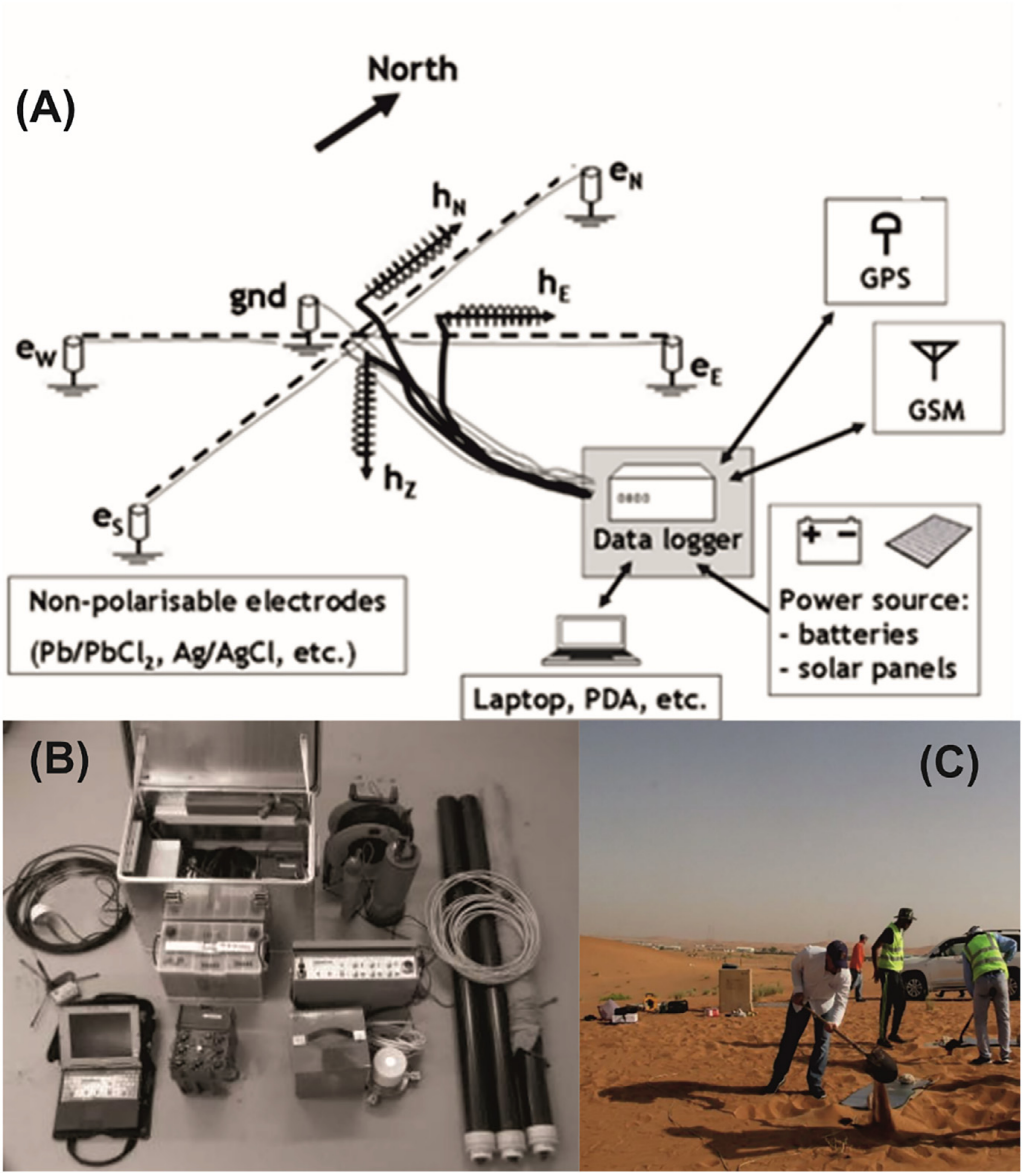
(A) Typical layout for a standard MT recording station. (B) Components of KMS-820 System. (C) Magnetotelluric survey in Al-Ain, June 2017.

**Figure 5:**
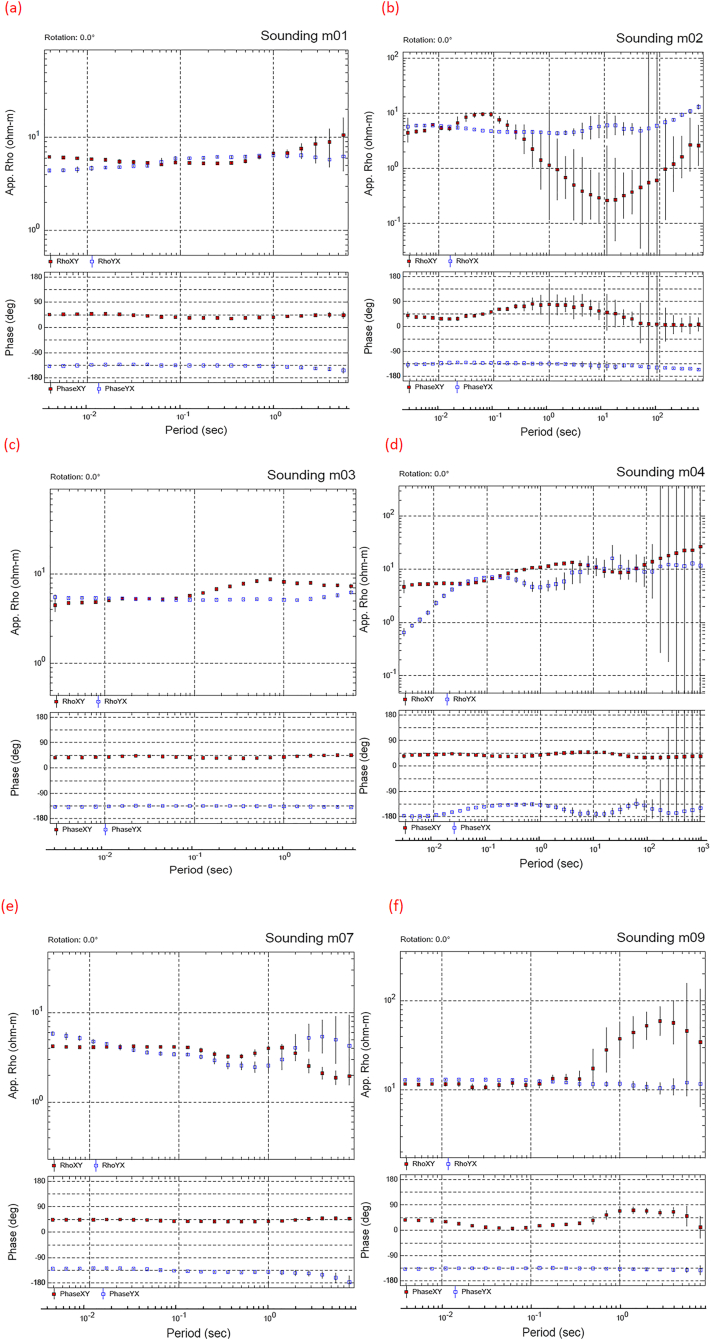
(a to f) Apparent resistivity and phase plots vs period for MT soundings m01, m02, m03, m04, m07 and m09. The red color represents the XY and the blue represent the YX components of the apparent resistivity values.

A profile, parallel to the Jabal Hafit (Hafit Mountain), consisting of nine stations (sites), was chosen for the MT measurements along the northern part of Hafit anticline at AMGA. A remote site was installed about 17 km away from the study area for better data quality (Figure 3). Figure 3 shows the location of the MT stations and the remote reference station (RR1). Data were recorded using a broad-band system (KMS-820 instrument from KMS Technologies) (Smirnov et al., 2008) (Figure 4). The recorded hours at each MT station is around 24 h. Most of the collected data quality was generally good, except for stations m05, m06 and m08, which were discarded due to their high noise levels. The apparent resistivity and phase plots vs period are shown in Figure 5.

### 3.2 Dimensionality analysis

Dimensionality analysis of MT data is a common procedure for inferring the main properties of the geoelectric structures of the subsurface, such as the strike direction or the presence of superficial distorting bodies, and enables the most appropriate modeling approach to be determined (Martí et al., 2010). Prior to forward modelling and inversion of the impedance data, it is important to have an understanding of the degree of distortion and dimensionality of the data sets. At the same time, it provides information on any effects of galvanic distortion on the data, so that data can then be appropriately corrected (Groom and Bailey, 1989; Smith, 1995). There are different approaches (dimensionality tools) proposed by different workers to check the dimensionality of MT data. In this paper, we used *phase tensor*, *Swift Skew* and *Bahr Skew* dimensionality tools to learn about the subsurface structure prior to inversion.

#### 3.2.1 Phase tensor analysis

Phase tensor (**Φ**) is defined as the ratio of the real (**X**) and imaginary parts (**Y**) of the complex impedance tensor, **Z** (Caldwell et al., 2004).

(3) **Φ** = **X**^−1^**Y**

**Figure 6:**
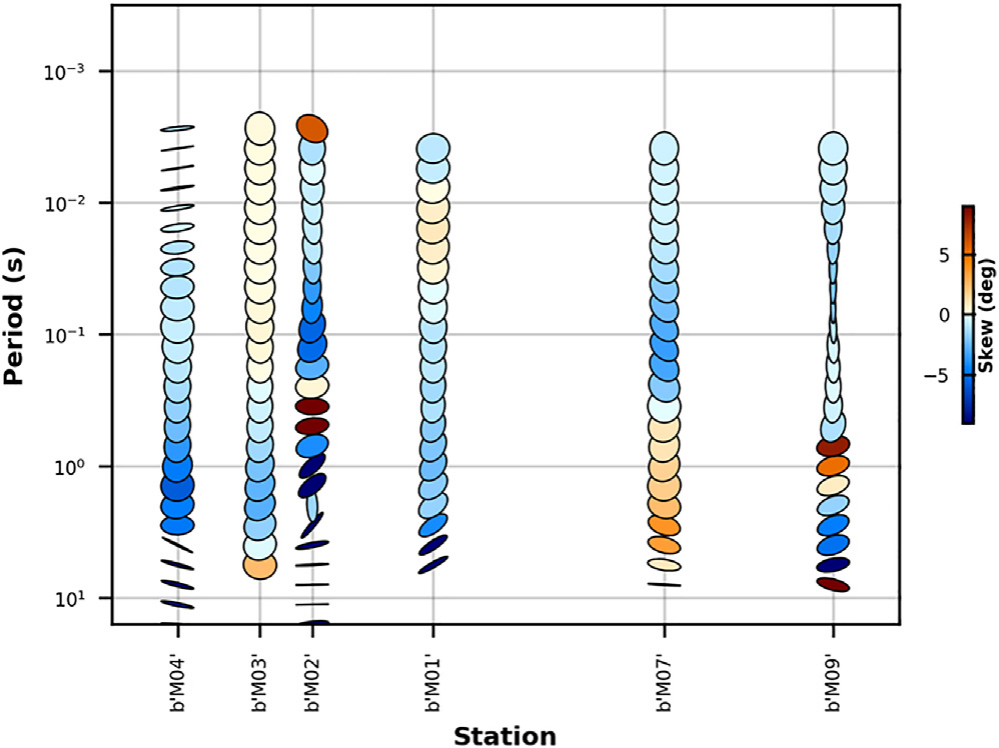
Phase tensor pseudo-section along the profile for recorded periods up to 10 s. The fill colors are the skew angles *β*. Most stations and period ranges show low skew values (∣*β*∣ < 3^o^), which justifies a 2-D inversion approach (Caldwell et al., 2004).

The phase tensor is a practical tool for easily obtaining information about the dimensionality of the regional structure (Caldwell et al., 2004; Cherkose et al., 2018). Phase tensor requires no assumption about the dimensionality of the underlying conductivity distribution and is applicable where the regional structure is 3-D. The phase tensor is represented graphically as an ellipse, with minimum (Φ min) and maximum (Φ max) principal axes, and skew angle (represented by the color in the ellipses) as shown in Figure 6. The skew angle (β) can be used for dimensionality analysis and its value is commonly displayed as color filling of the ellipses in phase tensor maps. For 1-D Earth (layered subsurface), the phase tensor is circular in shape and indicates a small skew angle. In the case of a 2-D regional resistivity structure, the phase tensor is characterized by an elliptical shape and β becomes small to zero in error-free data. In 3-D Earth, the phase tensor is non-symmetric and the skew angle (β) shows large values. A rapid lateral change of the principal axes of the phase tensor also indicates 3D structures (Caldwell et al., 2004).

**Figure 7:**
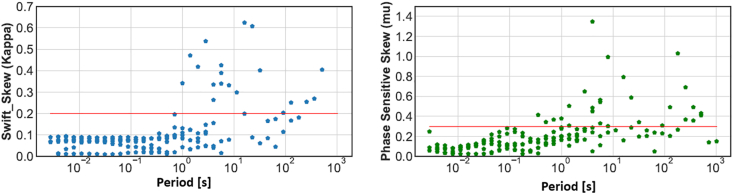
Swift skew is blue and Bahr skew in green plots for all 6 stations (m01, m02, m03, m04, m07 and m09). Most stations indicate 1D and 2D structures at short and longer periods in both the Swift skew and Bahr Skew analysis. Some stations indicate values above the thresholds (red line, 0.2 for the swift skew and 0.3 for the Bahr skew, respectively). This result is similar to the phase tensor analysis indicating generally a regional subsurface 1D and 2D structures.

In AMGA, the phase tensor pseudo-section plotted at different periods revealed the dimensionality of the subsurface structures (Figure 6). In general, our MT data have very small beta values and rounded ellipses (Figure 6). At short periods, the phase tensor indicates small skew angle (∣*β*∣ < 3^o^). The ellipses mostly are circular and elliptical shapes implying structures characterized as 1-D and 2-D. The low degrees of dimensionality, supported by the small Swift-Skew (Swift, 1967) and phase sensitive skew (Bahr, 1988, 1991) values for most of the periods calculated, allow for a two-dimensional (2-D) interpretation of the data (Figure 7). In Figure 7, in most cases the Swift-Skew from all the six stations denoted by the blue dots, indicate low values below 0.2 (represented by the red line). Similarly the Bahr skew (phase sensitive skew), represented by the green colored dots in the plot indicates low values below 0.3 in general. The results from both the Swift skew and Bahr Skew show similar results to the phase tensor analysis indicating regional 1D and 2D structures.

**Figure 8:**
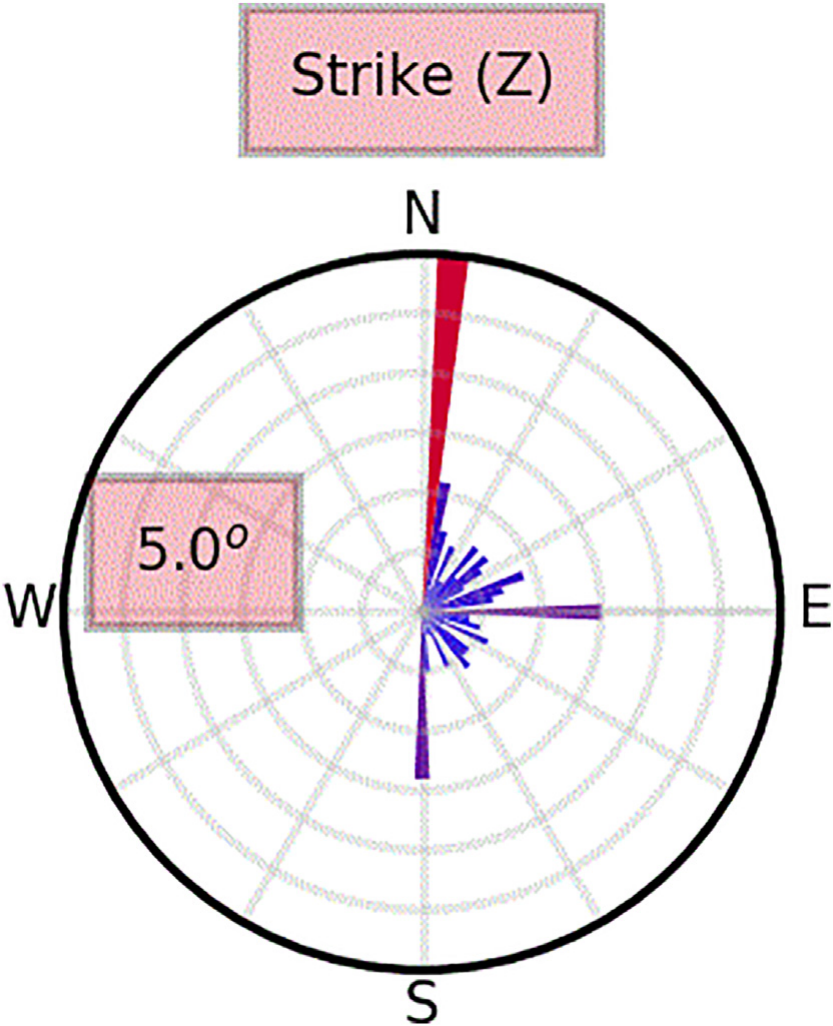
Rose diagram of strikes estimates (N5^o^E) for the Al-Mubazzarah Geothermal Area (AMGA) MT data dataset. The strike analysis is done for the entire frequency range.

In the case of a 2D earth, the strike direction must be found in order to decompose the impedance tensor into E and H polarizations. E-polarization (also referred to as TE mode) contains electric fields parallel to the strike of the lateral contrast as well as the perpendicular and the vertical component of the magnetic field, and B-polarization (also referred to as TM mode) includes the electric field component crossing the resistivity contrast and the corresponding magnetic field component. The regional strike direction was estimated by the **MTpy**, an open source python program (Krieger and Peacock, 2014). The N5^o^E strike estimates (Figure 8) are fairly consistent and close to parallelism with the major NNW-SSE fault system in AMGA, and are recognized as the regional strike direction. The impedance components were rotated N5^o^E before the 2-D inversion.

## 4 2D MT model

In this paper, we used a smooth model inversion routine of the software package WinGLink® by Geosystem based on Rodi and Mackie (2001). This routine finds regularized solutions (Tikhonov Regularization) to the two-dimensional inverse problem for Magnetotelluric data using the method of nonlinear conjugate gradients (NLCG). The forward model simulations are calculated using the finite difference method (FDM).

For our study area, the 2D model was generated with 6 MT stations aligned in an almost N-S direction. A joint 2-D inversion, which included both **TE** and **TM** modes was performed. The joint inversion of the **TE** and **TM** mode data was used in order to derive an overall picture of the subsurface conductivity structure in the AMGA area that would explain the data from both polarizations simultaneously. A total of 22 frequencies in the range 0.00276 Hz–255 Hz were used for the 2-D inversion. The data was rotated to the appropriate geoelectric strike direction prior to the inversion.

**Figure 9:**
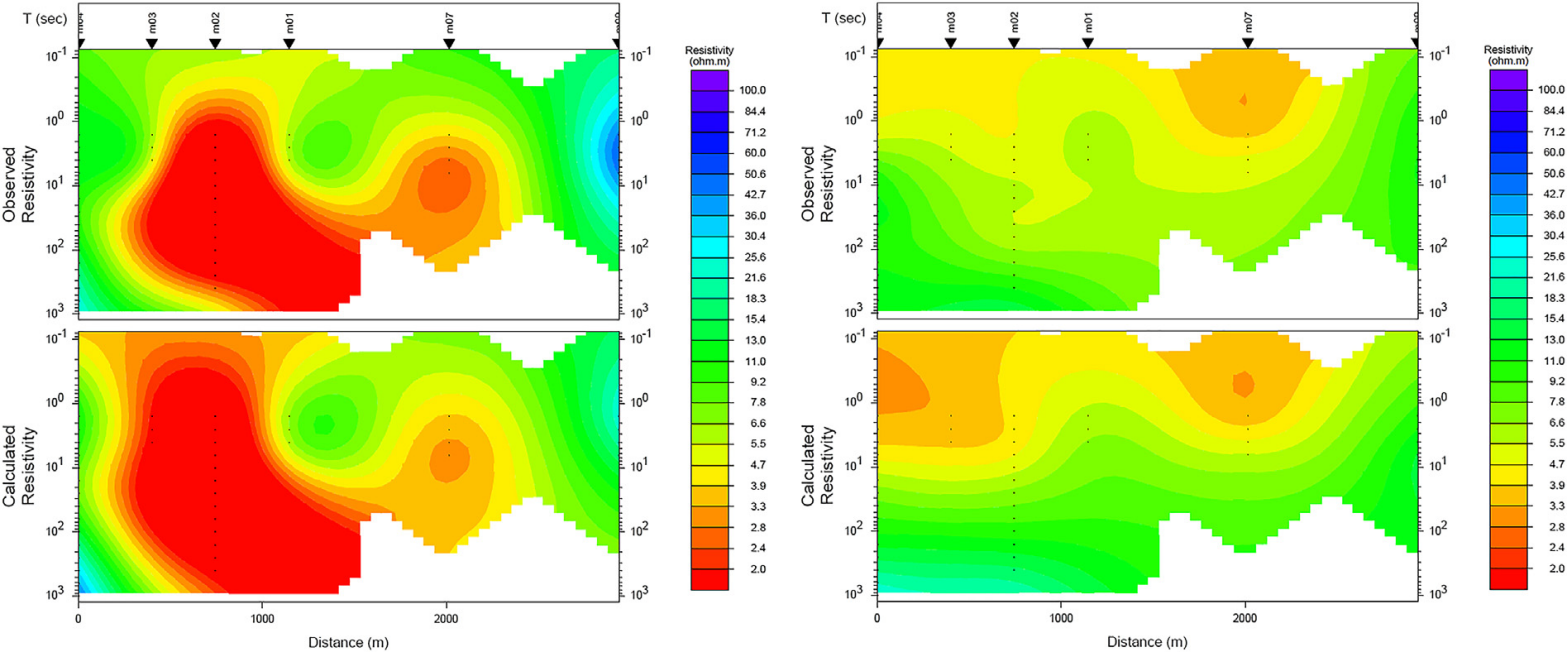
Observed apparent resistivity and calculated data of TE (left) and TM (right) modes for the preferred inversion mode. The final RMS is 2.413 after 30 iteration.

We used a large error floor for the apparent resistivity and a small error floor for the phase during inversion to accommodate the static shift. This approach is one technique to minimize the effect of static shift in the magnetotelluric data (Wu et al., 1993; Ogawa, 2002). Static shift caused by local near-surface inhomogeneity or topography, can result in the parallel shift of the apparent resistivity curves, while the phase curves are basically consistent. In the 2D model, the error floors for the apparent resistivity and phase were set to 15% and 5% for the joint (TM and TE) mode, respectively. Different error floors were tested to obtain a good fit between the observation and model response. For the inversion, a fine mesh was generated with a homogenous 15 Ωm as a starting initial model. The data misfit is presented with pseudosection plot of observed and predicted responses for the last iteration (Figure 9). The final RMS was 2.413 at the 30^th^ iteration.

## 5 Gravity and magnetic data

Potential field data (gravity and magnetic) were collected in the study area during 2017. The gravity data were acquired using Scintrex CG-6 gravity-meter and the data was corrected for tilt, tide, topography, location, drift and Bouguer density (Saibi et al., 2019, 2020) in order to calculate the Bouguer anomalies. The magnetic data were acquired using a Geometrix G-856 AX Proton magnetometer (Mohamed and Saibi, 2017; Saibi et al., 2017; Saibi, 2018). The recorded magnetic data was converted to Reduced to the Pole (RTP) using a Declination of 1.98^o^ and an Inclination of 38.17^o^ (Saibi, 2018). To understand the subsurface rock properties and structure, we inverted the potential field data using Petrel software (Trademark of Schlumberger Corporation) and extracted from the 3D potential field inversion model the results of a cross-section along the same MT stations for comparison and integration purposes. More details on the inversion methodology incorporated in Petrel software for potential field data are covered by Priezzhev and Pfutzner (2011).

## 6 Results and discussions

**Figure 10:**
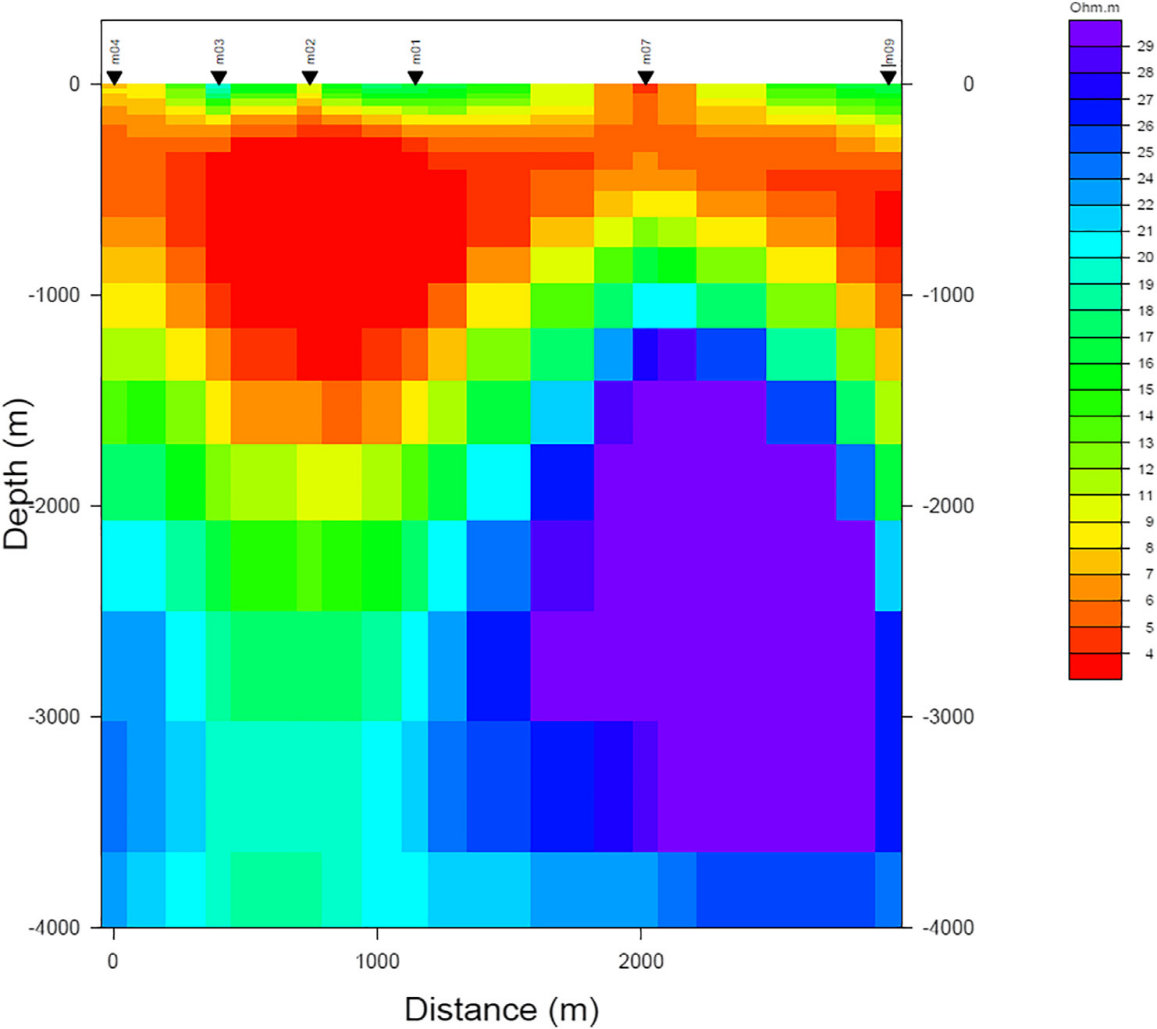
Resistivity cross-section obtained from 2D inversion of MT data. Shallow sediments were imaged as moderately resistive above a low resistivity layer followed by a gradually increasing resistivity zone.

The preferred 2-D model along the profile (to a depth of 4 km) is depicted in Figure 10. The model shows three distinctive resistivity layers. The first (uppermost) layer (1) is shallow and has resistivity ranging from 10 to 20 Ωm, representing the clastic sedimentary rocks (Miocene to Quaternary), which are a saturated geological layer (Murad et al., 2012). The second layer (2) is a conductive layer with resistivities less than 10 Ωm, lying beneath the first layer, and extending downwards for up to 2 km below stations m09 and m02. This conductive zone appears thinner near areas beneath station m07. This layer represents carbonate sequence (Eocene to Oligocene) which are common in this region. The third (lowermost) layer (3) is a deep moderate to high resistive zone, 10–30 Ωm extending downwards from 2 km to 4 km depth in northern part of the profile.

The conductivity of the second layer could be explained by the existence of saline water due to dissolution process happening within the carbonate formations. Hydrochemical studies of groundwaters from the study area showed very high Total Dissolved Solids (TDS) of the groundwater from Carbonate aquifer in Al-Ain ranging from 1,910 mg/L to 17,400 mg/L (Murad et al., 2011) and also could be related to conductive clay minerals, which are the cap rock of geothermal systems.

Resistivity distributions in a high-enthalpy geothermal field usually depend on clay minerals and temperature as well as formation lithology (Uchida and Sasaki, 2006; Uchida et al., 2005; Muñoz, 2014). In our survey area, however, temperature is not very high at the surveyed depths and hydrothermal clay minerals may not be abundant enough to significantly affect the resistivity distribution. Hence, the main purpose of our 2D MT model is to clarify the geometry of formation boundaries and major resistivity anomalies. However, the resistivity pattern observed in the study area corresponds very well to the expected resistivities in geothermal systems. Accordingly, the observed moderate to relatively high resistivity zone (10–30 Ωm) in the lower levels of the model may represent a region where the hot groundwaters originated (geothermal reservoir) with the hottest geothermal parts being located deeper than 4 km. This zone is interpreted as a probable geothermal reservoir hosted by late Cretaceous carbonates and conglomerates and underling metamorphosed igneous rocks. The depth to the probable geothermal reservoir is below 2 km up to 4 km, which is in agreement with calculated depths from geothermometry analyses of Saibi (2018). Saibi et al. (2020) estimated the temperature of the geothermal reservoir at AMGA of 147 °C using cation geothermometers. This result is very similar to other low enthalpy geothermal systems in sedimentary basins (e.g. Gross Schönebeck geothermal site in Germany (Muñoz et al., 2010).

**Figure 11:**
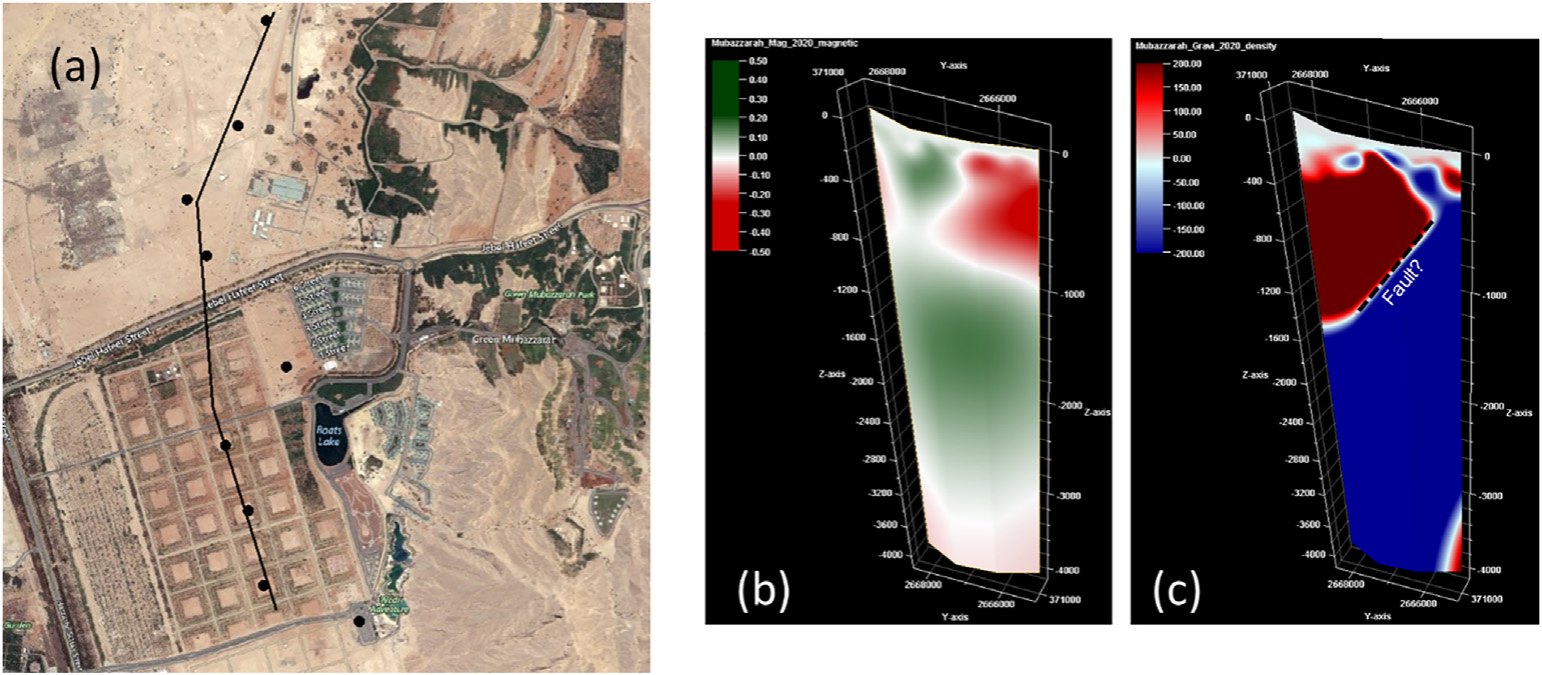
(a) Profile location used for dissecting inverted gravity and magnetic results from 3D potential field inversion, black dots are locations of MT stations. (b) is magnetic inversion (magnetic susceptibility in SI), (c) is gravity inversion (density contrasts in g/cm^3^). The gravity inversion cross-section show a possible normal fault dipping to the north at about 25^o^, or alternatively it represent a buried erosional surface (non-conformity). If it is a fault it could connect AMGA to the Ain Faidha hot springs, as the orientation of faults in this area is almost E-W. A probable fault is also observed from the resistivity cross-section calculated from MT data (see Figure 10).

The 2D resistivity model (Figure 10) agrees with fault dipping 58 ^o^, consistent with the 2D gravity inversion results (Figure 11c). The trend of the nearby faults and fractures is E-W to WNW-ESE, and these are mainly extensional structures (Zaineldeen and Fowler, 2014) that could provide a conduit for hot waters between the two hot springs occurrences (AMGA and Ain Faidha). E-W normal faults are the dominant fracture systems in the study area (Zaineldeen and Fowler, 2014; Styles et al., 2006).

The study area was covered by closely spaced gravity and magnetic measurements in order to better understand the subsurface structure. The details of the field measurements and corrections are presented in Saibi et al. (2019); Saibi et al. (2020); Saibi (2018); Mohamed and Saibi (2017); Saibi et al. (2017). Figure 11 shows the gravity and magnetic inversion results along the same traverse as that of the MT stations for comparison and better integration of the geophysical results from the study area. From Table 1, a resistivity variation with depth probably reflects decreasing salinity of groundwaters from near surface saline to deeper fresher waters. The density change from higher to lower with depth appears odd, but could be due to intensively fractured basement carbonate rocks relative to the less fractured cover sediments. The magnetic susceptibility increases with depth as expected, however the deeper part in the basement have slightly lower magnetic susceptibility due to high temperatures.

**Table 1:** Comparison between the three geophysical methods (MT, gravity, and magnetic) results.

Geological layer	MT (Ωm)	Gravity (density)	Magnetic (magnetic susceptibility)
A (clastic sediments)	10–20	Moderate	Moderate
B (carbonate sequence)	<10	High	Low to moderate
C (geothermal reservoir hosted in Mesozoic basement rock)	10–30	Low to high (mainly low)	Low to moderate

**Figure 12:**
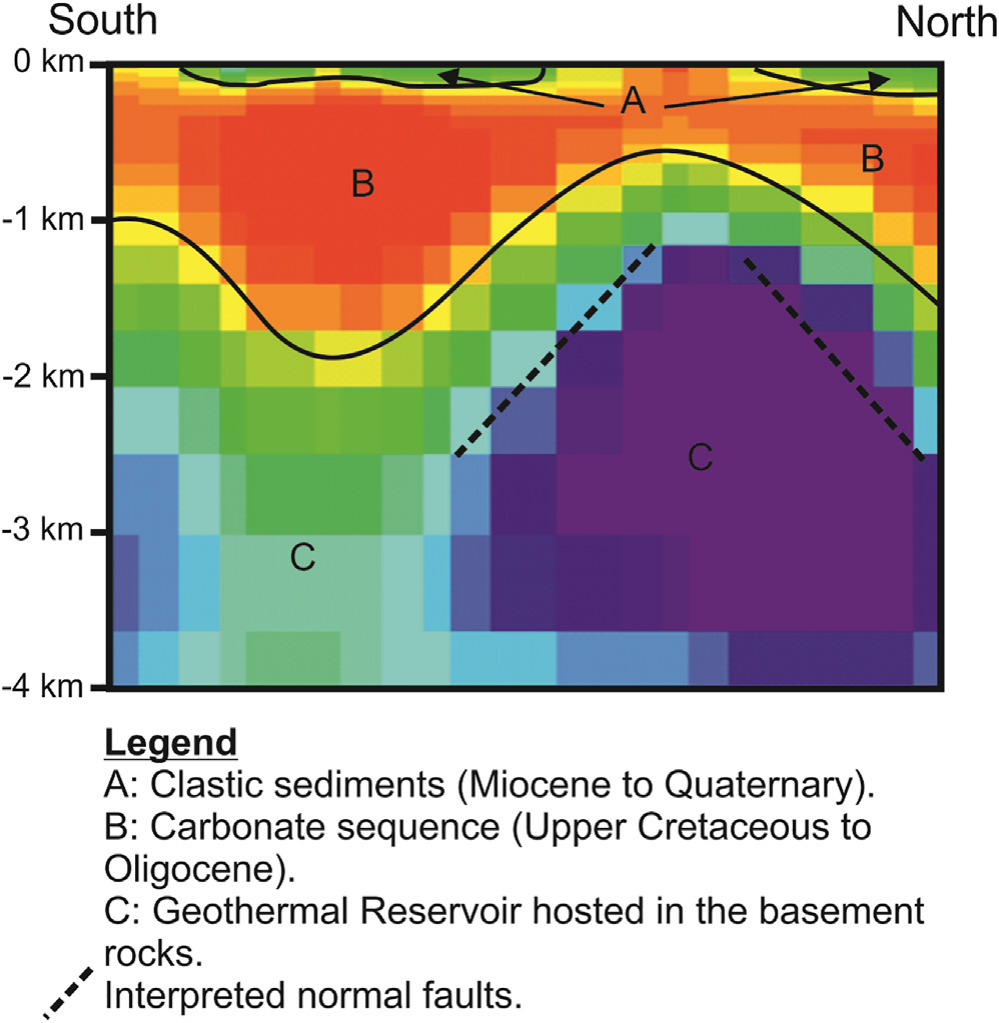
Interpretational geothermal-geological model of the study area from MT results.

Figure 12 shows the geological conceptual model of the study area from MT data consolidated with potential field results.

## 7 Conclusions

MT (Magnetotelluric) survey was conducted in 2017 along the northern part of Hafit anticline, southeast of Al Ain city, UAE to investigate the geothermal potential of the Al-Mubazzarah geothermal area. Preliminary resistivity structures were obtained by 2D inversions of the MT data. The 2D model indicated a shallow layer with a resistivity value ranging between 10 to 20 Ωm, associated with recent sediment deposits in the area. Beneath the first layer, a conductive layer with resistivities less than 10 Ωm was revealed by the 2D model. This conductive zone appears thinner near areas beneath station m07. This layer represents the Tertiary carbonate sequence which is common in this region. Beneath the second layer, a moderate to highly resistive zone, 10–30 Ωm represents Mesozoic basement rocks from 1 km and reaching up to 4 km in northern part of the profile. This zone is interpreted as a probable geothermal reservoir hosted by late Cretaceous carbonates and conglomerates and underling metamorphosed igneous rocks. This geothermal reservoir is also characterized by a low density and a high magnetic susceptibility. This study is a first attempt to investigate this geothermal area using MT and further studies are required to better understand the geothermal system in finer detail in the future.

## Declarations

### Author contribution statement

Hakim Saibi: Conceived and designed the experiments; Analyzed and interpreted the data; Contributed reagents, materials, analysis tools or data; Wrote the paper.

Sadieh Khosravi: Conceived and designed the experiments; Analyzed and interpreted the data; Wrote the paper.

Biruk Abera Cherkose, Yosef Kebede, Abdel-Rahman Fowler: Analyzed and interpreted the data; Wrote the paper.

Maxim Smirnov: Performed the experiments, Analyzed and interpreted the data.

### Funding statement

This research was funded by the Research Office of United Arab Emirates University (Research Start-Up Grant No. 8, Fund 31S264, 2016).

H. Saibi and B. A. Cherkose thank the UAEU for financing the numerical simulation of this research (Grant No. 31S394).

### Data availability statement

Data will be made available on request.

### Declaration of interests statement

The authors declare no conflict of interest.

### Additional information

No additional information is available for this paper.

## References

[bib2] Abdel Zaher M., Saibi H., Nishijima J., Fujimitsu Y., Mesbah H., Ehara S. (2012). Exploration and assessment of the geothermal resources in the Hammam Faraun hot spring, Sinai Peninsula, Egypt. J. Asian Earth Sci..

[bib3] Ali M., Sirat M., Small J. (2009). Integrated gravity and seismic investigation over the Jabal Hafit structure: implications for basement configuration of the frontal fold-and-thrust belt of the Northern Oman Mountains. J. Petrol. Geol..

[bib5] Amatyakul P., Boonchaisuk S., Rung-Arunwan T., Vachiratienchai C., Wood S.H., Pirarai K., Fuangswasdi A., Siripunvaraporn W. (2016). Exploring the shallow geothermal fluid reservoir of Fang geothermal system, Thailand via a 3-D magnetotelluric survey. Geothermics.

[bib6] Bahr K. (1988). Interpretation of the magnetotelluric impedance tensor: regional induction and local telluric distortion. J. Geophys..

[bib7] Bahr K. (1991). Geological noise in magnetotelluric data: a classification of distortion types. Phys. Earth Planet. In..

[bib8] Boote D.R.D., Mou D., Waite R.I. (1990). Structural evolution of the Suneinah foreland, Central Oman mountains. Geol. Soc. Lond., Spec. Publ..

[bib9] Caldwell T.G., Bibby H.M., Brown C. (2004). The magnetotelluric phase tensor. Geophys. J. Int..

[bib10] Cherkose B.A., Mizunaga H., Samrock F. (2018). Imaging resistivity structures of high-enthalpy geothermal systems using magnetotelluric method: a case study of Aluto-Langano geothermal field in Ethiopia. Proceedings, 7th African Rift Geothermal Conference, Kigali, Rwanda 31st October – 2nd November 2018.

[bib11] Cherkose B.A., Mizunaga H. (2018). Resistivity imaging of Aluto-Langano geothermal field using 3-D Magnetotelluric inversion. J. Afr. Earth Sci..

[bib12] Chiragwile S.A. (2009). Interpretation of resistivity soundings in the Krusuvik high-temperature geothermal area, SW-Iceland, using joint inversion of TEM and MT data. Geothermal Training Programme.

[bib13] Dunne L.A., Manoogian P.R., Pierini D.F. (1990). Structural style and domains of the northern Oman mountains (Oman and United Arab Emirates). Geol. Soc. Lond., Spec. Publ..

[bib14] Erdoğan E., Candansayar M.E. (2017). The conductivity structure of the Gediz Graben geothermal area extracted from 2D and 3D magnetotelluric inversion: synthetic and field data applications. Geothermics.

[bib16] Glennie K.W. (1974). Geology of the Oman Mountains.

[bib17] Groom R.W., Bailey R.C. (1989). Decomposition of magnetotelluric impedance tensors in the presence of local three-dimensional galvanic distortion. J. Geophys. Res. (Solid Earth).

[bib18] Hersir G.P., Árnason K. (2009). Resistivity of Rocks.

[bib19] Krieger L., Peacock J.R. (2014). Mtpy: a Python toolbox for magnetotellurics. Comput. Geosci..

[bib20] Martí A., Queralt P., Ledo J., Farquharson C. (2010). Dimensionality imprint of electrical anisotropy in magnetotelluric responses. Phys. Earth Planet. In..

[bib21] Mohamed H., Saibi H. (2017). 3-D forward modelling of magnetic data from Al-Mubazzarah geothermal field, Al-Ain, United Arab Emirates. The 4th International Conference on Engineering Geophysics (ICEG), Al-Ain, UAE, 9-12 October 2017.

[bib22] Mohan K., Kumar G.P., Chaudhary P., Choudhary V.K., Nagar M., Khuswaha D., Rastogi B.K. (2017). Magnetotelluric investigations to identify geothermal source zone near Chabsar hotwater spring site, Ahmedabad, Gujarat, Northwest India. Geothermics.

[bib23] Mohan K., Chaudhary P., Pavan Kumar G., Rastogi B.K. (2019). Magnetotelluric investigations in the southern end of the Cambay basin (near coast), Gujarat, India. J. Appl. Geophys..

[bib24] Muñoz G. (2014). Exploring for geothermal resources with electromagnetic methods. Surv. Geophys..

[bib25] Muñoz G., Ritter O., Moeck I. (2010). A target-oriented magnetotelluric inversion approach for characterizing the low enthalpy Groß Schonebeck geothermal reservoir. Geophys. J. Int..

[bib26] Murad A., Mahgoub F., Hussein S. (2012). Hydrogeochemical variations of groundwater of the northern Jabal Hafit in eastern part of Abu Dhabi Emirate, United Arab Emirates. Int. J. Geosci..

[bib27] Murad A.A., Gerish M.H., Mahgoub F.M., Hussein S. (2011). Physiochemical processes affecting the geochemistry of carbonate aquifer of southeastern Al-Ain area, United Arab Emirates (UAE). Water Air Soil Pollut..

[bib28] Noweir M.A. (2000). Back-thrust origin of the Hafit structure, northern Oman mountain front, United Arab Emirates. GeoArabia.

[bib29] Ogawa Y. (2002). On two-dimensional modeling of magnetotelluric field data. Surv. Geophys..

[bib30] Priezzhev I., Pfutzner H. (2011). US Grant 8700372B2: Method for 3-D Gravity Forward Modeling and Inversion in the Wavenumber Domain.

[bib31] Rodi W., Mackie R.L. (2001). Nonlinear conjugate gradients algorithm for 2-D magnetotelluric inversion. Geophysics.

[bib32] Saibi H. (2018). Various geoscientific investigations of low-enthalpy geothermal sites in the United Arab Emirates. Proceedings 43rd Workshop on Geothermal Reservoir Engineering.

[bib33] Saibi H., Gabr A., Baker H., Al Bloushi K. (2017). 3-D magnetic inversion at Al-Mubazzarah area, Al-Ain, United Arab Emirates. The 4th International Conference on Engineering Geophysics (ICEG), Al-Ain, UAE, 9-12 October 2017.

[bib34] Saibi H., Batir J., Pocasangre C. (2020). Hydrochemistry and geothermometry of thermal waters from UAE and their energetic potential assessment. Geothermics.

[bib35] Saibi H., Amrouche M., Fowler A. (2019). Deep cavity systems detection in Al-Ain city from gravity surveys inversion. J. Asian Earth Sci..

[bib36] Saibi H., Amrouche M., Batir J., Pocasangre C., Hussein S., Gabr A., Aldahan A., baker H., Nishijima J., Gottsmann J. (2021). 3D geologic model and energy potential estimation of UAE Geothermal Systems Mubazzarah-Ain Faidha and Ain-Khatt. World Geothermal Congress, 2021.

[bib37] Searle M.P., Cooper D.J.W., Watts K.F., Robertson A.F.H., Searle M.P., Ries A.C. (1990). Structure of the jebel Sumeini – jebel Ghawil area, northern Oman.

[bib38] Simpson F., Bahr K. (2005). Practical Magnetotellurics.

[bib39] Smirnov M., Korja T., Dynesius L., Pedersen L.B., Laukkanen E. (2008). Broadband magnetotelluric instruments for near-surface and lithospheric studies of electrical conductivity: a fennoscandian pool of magnetotelluric instruments. Geophysica.

[bib40] Smith J.T. (1995). Understanding telluric distortion matrices. Geophys. J. Int..

[bib41] Styles (2006). The Geology and Geophysics of the United Arab Emirates.

[bib42] Swift C.M. (1967). A Magnetotelluric Investigation of Electrical Conductivity Anomaly in the Southwestern United States. http://hdl.handle.net/1721.1/38346.

[bib43] Uchida T., Sasaki Y. (2006). Stable 3D inversion of MT data and its application to geothermal exploration. Explor. Geophys..

[bib44] Uchida T., Song Y., Lee T. (2005). Magnetotelluric survey in an extremely noisy environment at the Pohang low-enthalpy geothermal area, Korea. Proc. World Geotherm. Congr..

[bib45] Wu N., Booker J.R., Smith J.T. (1993). Rapid two-dimensional inversion of COPROD2 data. J. Geomagn. Geoelectr..

[bib46] Zaineldeen U., Fowler A. (2014). Structural style and fault kinematics of the lower Eocene Rus Formation at Jabal Hafit area, Al Ain, United Arab Emirates (UAE). Arab. J. Geosci..

